# Slope with Predetermined Shear Plane Stability Predictions under Cyclic Loading with Innovative Time Series Analysis by Mechanical Learning Approach

**DOI:** 10.3390/s22072647

**Published:** 2022-03-30

**Authors:** Tingyao Wu, Hongan Yu, Nan Jiang, Chuanbo Zhou, Xuedong Luo

**Affiliations:** 1 Faculty of Engineering, China University of Geosciences (Wuhan), Wuhan 430074, China; wutingyao@cug.edu.cn (T.W.); cbzhou@cug.edu.cn (C.Z.); cugluoxd@foxmail.com (X.L.); 2 CCCC Second Highway Consultants Co., Ltd., Wuhan 430056, China; hh5-106yha@163.com

**Keywords:** slip zone soils, cyclic loading, strain softening, fracture zone, landslide triggering mechanisms, mechanical learning

## Abstract

We propose a mechanical learning method that can be used to predict stability coefficients for slopes where slopes with predetermined shear planes are subjected to cyclic seismic loads under undrained conditions. Firstly, shear tests with cyclic loading of different parameters were simulated on designated slip zone soil specimens, in which the strain softening process leading to landslide occurrence was closely observed. At the same time, based on the limit equilibrium analysis of the Sarma method, the variation of slope stability coefficients under different cyclic loads was investigated. Finally, a Box–Jenkins’ modeling approach is used to predict the data from the time series of slope stability coefficients using a mechanical learning approach. The simulation results show that (1) reduction in coordination number can be an accurate indicator of the level of strain softening and evolutionary processes; (2) the gradual reduction of shear stress facilitates the soil strain softening process, while different cyclic loading stress amplitudes will result in rapid penetration or non-penetration of the fracture zone by means of particulate flow. Although the confining pressure of the slip zone soil can inhibit the increase of fractures, it has a limited inhibitory effect on strain softening; (3) based on field observations of the slope stability factor and stress field, two possible landslide triggering mechanisms are described. (4) Mechanical learning of time series can accurately predict the changing pattern of stability coefficients of slopes without loading. This study establishes a potential bridge between the geological investigation of landslides and the theoretical background of landslide stability coefficient prediction.

## 1. Introduction

Landslides are geological phenomena that can occur on land, but also under the seabed due to earthquakes, tsunamis, etc. At the same time, they include various types of movements, such as slope failures, rock falls, or mudslides [1]. Landslide were also defined as a mass of rock, debris, or earth, moving or sliding down a slope [2]. Landslides are one of the most frequent geological hazards. It has long been believed that one of the main triggers of landslides is earthquakes, which can induce large-scale and catastrophic landslides with the loss of human lives or property damage [3,4]. According to statistics for many countries, landslides cause serious social and economic impacts globally, for example, between 2007 and 2015, more than 7200 landslides were recorded worldwide, causing more than 26,000 deaths and costing more than $1.8 billion in damages [5]. Meanwhile, Japan is known to contain a frequency of catastrophes, such as earthquakes, volcanic eruptions, landslides, and typhoons [6,7,8,9,10,11]. According to the Japan Meteorological Agency (JMA), 230 aftershocks occurred between 6 September and 11 September 2018, killing over 44 people and injuring over 660 [12]; this also had a significant impact on Hokkaido’s infrastructure, causing a combined economic loss of US$2 billion in damage to transportation and utilities [13]. In addition, the transboundary Koshi River basin, located in the central Himalayas, is shared by China, Nepal and India. The triggering mechanism and landslide number were counted based on remote sensing findings, providing information that 5858 rainfall-triggered landslides occurred in the study area between 1992 and 2015, and an additional 14,127 seismic landslides were mapped after the 2015 Gorkha earthquake [14]. In China, landslides are the second most frequent natural cause of damage to man-made structures after earthquakes, so it is foreseeable that the potential increase in the number of extreme weather events, combined with the concentration of population and infrastructure in mountainous areas, will lead to an increase in landslide-related casualties in the future [15,16,17]. A better understanding of landslide triggering mechanisms and monitoring the early movement of soil mass, along with effective evacuation strategies as early as possible, are essential for landslide mitigation [18]. 

Landslides are related to different factors, such as topography, geology, tectonic history, weathering erosion history and land use. However, landslides are usually considered to be triggered by only one factor [19]. A trigger is considered to be an external stimulus, such as a strong rainfall event, an earthquake of different magnitudes, a volcanic eruption, a storm, or rapid flow in the form of erosion leading to a rapid increase in stress or strain on the landslide and a decrease in the slip zone soil material [19,20,21]. Based on classical elastoplastic mechanics theory, the strain softening process is reduced to a series of brittle plasticity processes, and thus the solution for the strain softening problem is reduced to the solution of a series of brittle plasticity problems, also, the strain softening behavior is the result of the soil reaching the peak stress point and then the stress decreasing as the strain continues to increase, i.e., the strength of the soil decreases with increasing strain, as a result of the spatial rearrangement of the soil particles and the forces between the phases and particles in the soil [22]. Moreover, higher cyclic stress ratios accelerate the softening behavior of soils, and different super consolidation ratios have a great influence on the softening of soils [23,24,25], and changes in the principal stress direction can cause structural remodeling of clays, which leads to a reduction in clay strength. Therefore, it is essential to investigate the strain softening characteristics of soil under cyclic loading. Examining the rupture mechanism is necessary if the stress state of the soil wants to be understood, especially the characteristics of the particle arrangement inside the soil. However, it is not practical to directly measure the soil distribution characteristics, especially the soil particle arrangement. Considering the close relationship between stress and strain, the majority of laboratory experiments have specialized in the characterization of displacements. Mounting strain gauges and displacement gauges on the surface of the device loaded with soil material are the most straightforward and simplified methods for measuring stress and deformation in laboratory tests. There are also some parts that use non-touch optical technology, such as electronic photography and other related technologies [26,27]. However, the placement of CT is somewhat limited due to the three-dimensional stress state and difficult exposure of the slip zone soils [28,29]. The main point to be made is that stress-strain relationships in soils are often full of randomness and strongly depend on the stress state and structural characteristics of the various materials. Although the empirically determined principal structure equations can be easily derived from the stress-strain values of soils, they are sometimes questionable.

Meanwhile, in the study of landslide mechanisms, typically landslide trigger and formation mechanisms are studied by various methods, such as traditional field surveys, satellite remote sensing, and three-dimensional imaging by drones. Numerical simulations and model experiments have also been used, but the studies have focused on macroscopic and microscopic parameters for the analysis. As in field testing, regarding the characterization of landslide studies, size and velocity, depth, impact pressure, or displacement all play an important role, and different types of mass movements are different. Volume may be a more important landslide feature than size, but this is difficult to measure because it requires specific geophysical or geotechnical methods. In order to solve the above-mentioned problems, researchers generally also go through numerical simulation software to study the triggering mechanism of landslides, for example, there are now two main methods for numerical simulation of landslides: continuum (finite element) and discontinuity (discrete unit). However, most previous studies have focused on the analysis of slope stability or dynamic response characteristics. To our knowledge, no attempt has been made to use PFC3D for inverse analysis of the microscopic mechanisms of landslides. Although earthquakes are the most important landslide causative factor, the relationship between cyclic microseismic loading and landslides has rarely been analyzed in detail, and no specific vibration control thresholds have been identified for specific shallow landslides [30,31]. At the same time, the precise onset of landslide damage is often unknown. In addition, the most destructive landslides are usually those caused by damage associated with deep-seated landslides. For such landslides, the relationship between cyclic micro seismic loading parameters and landslide occurrence is very complex [32,33]. 

With regard to the safety protection of slopes, the first thing to do is to clarify the safety state of the slope, but most of the existing research tools are used to obtain the stability state of the slope through the traditional quantitative solution approach, which has certain limitations. The safety of slopes contains many parameters and randomly variable laws, which leads to a relatively complex calculation process, and with the rapid rise of machine learning technology, a new way of thinking is proposed for the study of slope safety and stability. Decision trees [34], random forests, support vector machines [35,36,37] and plain Bayesian [38] algorithms have been widely used in slope research. By collecting and analyzing 250 slope data, 31 variables were identified and their functional relationships were explained with the help of Principal Component Analysis (PCA), thus constructing an algorithmic model to assess the stability of slopes with good generalization to the test data [39]. The slopes of the Klang Valley were studied with the help of a back-propagation neural network and calculated the weights of 11 relevant influencing factors, such as slope rate and slope height [40]. Genetic algorithms have been utilized to study specific problems in geotechnical slopes [41]. Multiple logistic regressions were carried out, while various methods, such as random forests, K-nearest neighbors’ algorithms and decision trees were also used to explore the steady state of the slopes [42].

However, various machine learning tools have emerged, mainly artificial neural networks and multiple regression analysis as the two classical models of machine learning, which have been widely used in solving various prediction problems. However, the weight analysis of the influence factors of these two models is not comprehensive enough, lacking a comprehensive comparison between the weight analysis based on correlation tests and the weight analysis based on intelligent models, while not considering the randomness and periodicity of time series data, resulting in large errors in forecasting.

Therefore, based on the work of Wong et al. (1998) and Zhang et al. (2021), the focus of this study is on the quantification of damage extent and estimation of shear stress in slip zone soils. Then, the effect of different parameters on the shear strain-softening behavior of the slip zone soils can be investigated, including the cyclic loading parameters (cyclic loading stress amplitude and the number of cyclic loading) and the confining pressure applied to the numerical soil model. The relationship between shear stress and soil fine-scale damage variables will also be discussed in detail in the paper. Meanwhile, comparing the results of discrete element numerical simulations with the changes in slope stability coefficients, the influence of simulation results on landslide formation will be interpreted. Finally, in combination with the Box–Jenkins modeling approach, the stability of slopes under different cyclic loads are systematically and comprehensively predicted from time series data of slope stability coefficients using a mechanical learning approach.

## 2. Numerical Methodology

### 2.1. Conceptual Model for Cyclic Shear Failure Behavior in Slip Zone Soils

**Figure 1:**
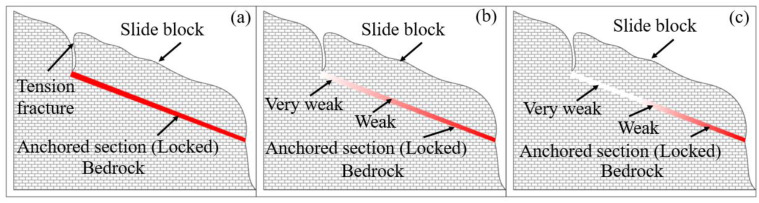
Evolutionary model of advancing layered rock landslide: (**a**) undamaged stage; (**b**) initial stage of damage; (**c**) end stage of damage.

The simulated situation can be interpreted as an advancing layered rock landslide, where the deformation is progressive from the trailing edge of the landslide to the front of the level flattening type cascading rock landslide, meanwhile, a detailed scheme for the evolutionary model of advancing layered rock landslides is presented in the specific work [43,44], as shown in Figure 1, where Figure 1 is a simplified model of a conceptual mechanics model that is designed to give the reader a more intuitive understanding of the landslide triggering mechanism. The gray grid in Figure 1 is the slope surface and bedrock, and the red part is called the anchored section of the landslide, which is the slip zone soil not affected by cyclic loading, but as the landslide evolution stage develops, the red part gradually becomes white, which means that the slip zone soil is affected by cyclic loading and its mechanical strength gradually weakens, and the unaffected part of the slip zone soil gradually becomes less and the safety factor of the landslide gradually decreases, that is, progressively enlarging the weakened upper zone while reducing the size of the lower locking block. The causes of landslides are mainly due to the strain softening rate of the slip zone soil at the trailing edge of the landslide under the cumulative effect of the cyclic loading. Moreover, under the action of long-term tension stress, deep and large tension fractures are produced at the trailing edge of the landslide. The mechanical properties of the trailing edge of the landslide are weaker than that of the leading edge of the landslide, and there is a phenomenon of gradually spreading development from the trailing edge to the leading edge, which means that the trailing edge of the landslide is destroyed first and the leading edge of the landslide is destroyed later. The process of landslide being triggered by cyclic loading has the characteristics of the trailing edge of landslide extruding to the leading edge of the landslide and locking of the leading edge. The evolutionary development of the advancing landslide can be divided into the following stages: (i) tension fractures are formed under the cumulative effect of the cyclic loading, and the fractures develop from the slope to the depth until they cut the underlying soft interlayer, and the tension fractures develop at the trailing edge of the landslide (Figure 1a); (ii) The mechanical structure of the soil in the slip zone at the trailing edge of the landslide is gradually destroyed, and the mechanical strength of the slip zone soil gradually decreases. As the landslide evolution advances, the weakening zone of the slip zone soil also gradually expands toward the leading edge (Figure 1b); (iii) with the expansion of the weakening zone, the length of the landslide locking section decreases accordingly. When the weakening zone expands to a certain length and the landslide resistance is insufficient to resist the sliding force of the landslide, the locking section is sheared out and the landslide is triggered along the penetrating slip surface (Figure 1c).

Based on the assumption of cyclic shear failure behavior, the conceptual model has to be able to (i) allow the rupture of interparticle links and (ii) characterize the microstructural features of the soil. For the first aspect, the discrete element method (DEM) was used considering its unique ability to characterize microfractures. The most commonly used DEM model for simulating soil strain softening is the linear Parallel Bond Model (PBM), the linear PBM includes two classical interface behaviors: a linear-elastic model interface containing only forces and a viscoelastic model interface containing both forces and moments. In the first interface, rotation is not applicable and only one direction of motion can be performed according to the forces, while the second model interface can accommodate the rotational action of the forces and also the motion under the forces, while the second model interface is automatically transformed into the first model interface when the forces between the particles exceed the defined yield value of the connection bonds. Meanwhile, the basic object studied by PFC3D is the contact between particles and particles, which can directly simulate the physical problems of motion and interaction between particles. The large particles of arbitrary shapes can be created by connecting two or more small particles, and the combined particles made by the connection can be studied as independent particle bodies. It avoids the study of mechanical properties of materials by obtaining an intrinsic model from traditional empirical data while studying the mechanical behavior of bulk media from a fine-scale perspective, and the method overcomes the macroscopic continuity assumption of traditional mechanical models of continuous media [45]. The engineering properties of soils are simulated numerically at the fine view level and the macroscopic mechanical behavior is analyzed by using the study of fine view parameters.

**Figure 2:**
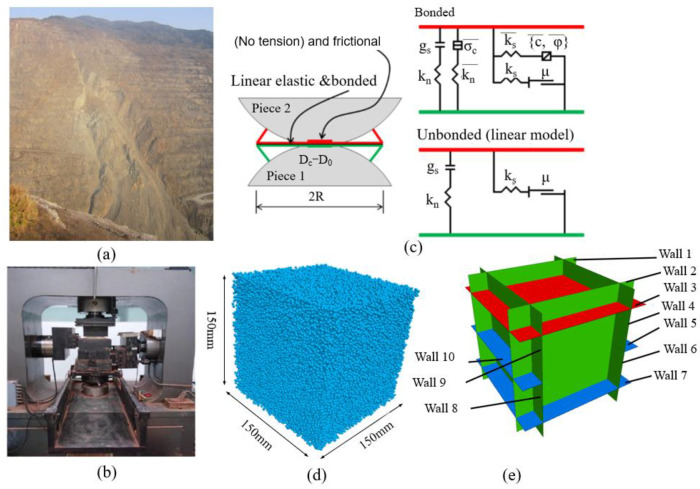
Concept of observing strain softening behavior tests and numerical modeling of slip zone soils at laboratory scale: (**a**) the slope with slip zone soil; (**b**) laboratory direct shear test; (**c**) linear Parallel Bond Model (Table 1 shows the meaning of the symbols of the parameters in the model); (**d**) numerical model of slip zone soil; (**e**) numerical model of walls for applying servo-control stress.

For this purpose, the slip zone soil fabrics are prepared and then covered with the base PBM, the grain boundary properties are simulated by modifying the intra-grain contact (see Figure 2c), and the length of the discrete microfractures are described by the midpoint of the line segment connected by two centers of mass and the average of their radii, respectively. By applying different types of cyclic loading to the numerical soil model, the accumulation of micro-fractures in the soil gradually forms macro-fractures, which eventually lead to shear failure damage of the slip zone soil due to soil strain softening. Therefore, it is promising to use this PBM to study the interaction and connection of microfractures under the action of cyclic loading. In order to consider the mechanical properties of shear failure of slip zone soil under cyclic loading, a detailed scheme for numerical modeling of slip zone soils is proposed. The concept of observing soil strain softening behavior tests and numerical modeling of slip zone soils at a laboratory scale is shown in Figure 2. The size of the discrete element model of slip zone soil is 150 mm × 150 mm × 150 mm (X∗Y∗Z), which means it is a tiny unit in the slip zone soil. The purpose of this selection is to analyze the variation of shear strength of slip zone soil particles under different dynamic loads from a fine viewpoint. Table 1 lists the calibrated microparameters of the slip zone soils. Slip zone soil was collected from an open pit of mine in Tieshan District, Huangshi City, Hubei Province, China.

### 2.2. Measurement Indicators- Coordination Number

The numerical simulation software PFC3D contains a set of more effective statistical methods to record the changes in variables between particles within the numerical model throughout the numerical simulation process, such as the forces and deformations, as well as the development of fractures [46]. One advantage of DEM simulations is the ability to obtain information that cannot be acquired from continuum-based techniques or physical experiments such as fabric analysis. In this regard, fabric tensor and coordination number provide a global description of contact orientations and packing stability. This criterion was used to assess the onset of instability (liquefaction) as loading progressed. Assuming that there are *N* particles in the measurement area, the coordinate number *C_n_* is defined as the average number of active contacts per body, and is computed as [47]:

(1) Cn=∑NncN

where *n_c_* is the number of contacts per particle. Once the contact force between the particles exceeds the limit value defined before the numerical calculation, then the contact between the particles will break due to the excessive force, followed by microscopic fractures being generated, while the number of contacts between the particles will be further reduced and the number of defined coordinates will gradually decrease. The decrease of coordination number implies breakage of particle contacts, which is called a fracture. 

### 2.3. Verification of the Accuracy of the Numerical Model and Details of the Applied Cyclic Loading 

The numerical simulation test process for slip zone soil materials consists of three parts: (i) stress initialization of the numerical model, (ii) servo control of the numerical model in predetermined confining pressure, and (iii) implementation of cyclic loading.

#### 2.3.1. Stress Initialization of the Numerical Model

The specimen consists of several particles, and after generating the particles, the particles and the wall are attributed with their own mechanical properties, and simultaneously with the connection properties between the particles and between the wall and the particles. Then, a certain porosity is attributed between the particles, and the particles are allowed to adjust freely to reach an equilibrium state without confining stress, i.e., stress initialization of the numerical model.

#### 2.3.2. Servo Control of the Numerical Model in Predetermined Confining Pressure

Following the generation of the sample, a servomechanism was applied iteratively to isotropically consolidate the specimen to the desired confining stress. The servomechanism uses the feedback of the stresses on the walls to determine if the isotropic stress is more or less than the desired value and adjusts the wall positions accordingly (Itasca, 2014). 

#### 2.3.3. Implementation of Cyclic Loading

Seismic waves generated by blasting are random waves, and it is better simulated if the original waveform is fed into the numerical calculation model for blasting vibration. However, because of the difficulty of frequency conversion and amplitude variation during the numerical test, all sinusoidal waveforms were used for the input vibration waveforms in the numerical test. In addition, in order to better compare the effects of amplitude and number of cycle loads on slope stability, simple waves were used for all studies in the full paper. At the same time, sinusoidal waves have most of the properties that seismic waves have and also have the advantage of simplifying calculations [48,49,50]. Therefore, after the consolidation phase, the sample was subjected to a strain-controlled sinusoidal cyclic loading pattern. During loading, the volume of the sample was conserved to simulate undrained conditions. The implementation of cyclic loading applied to the numerical model is the key to this simulation. Cyclic loading in laboratory tests is mainly controlled by inputting vibration waves to the test apparatus, while in PFC3D, it is mainly through the definition of wall velocity to achieve cyclic shear loading, which is essentially a displacement-controlled loading method. In the PFC3D simulation, the stress values between the loading wall and the particles are monitored to obtain the shear stress in the soil during cyclic loading. Based on the vibration parameters in the numerical model, a direct shear test is conducted mainly by the lower shear box, and a sine wave is an input to adjust the motion direction of the wall in real time, to achieve cyclic loading of the slip zone soil material. That is, the same shear rate is specified for walls 1#, 2#, 3#, 4#,5#, 9# and 10# in Figure 2. Figure 2 shows a schematic diagram of the cyclic loading process of the numerical model. During the numerical simulation, the PFC3D program mainly includes the measurement of the numerical model shear stress and the application of the wall velocity during cyclic loading. As shown in Figure 2 for the shear box, the force on the lower shear box is the sum of the contact force on wall 8 and the contact force on wall 6, while the force on the upper shear box is the sum of the contact force on wall 9 and the contact force on wall 4, and the number of cycle loading include 750, 1500, 2250, 3000, 3750, 4500, 5250, 6000, 7500, 9000, 11,500, 12,000, and cyclic loading stress amplitude include 0.5 cm/s, 0.9 cm/s, 1.5 cm/s, 2.2 cm/s, where cyclic loading stress amplitude is equivalent to the peak ground velocity (PGV) of a natural earthquake.

**Figure 3:**
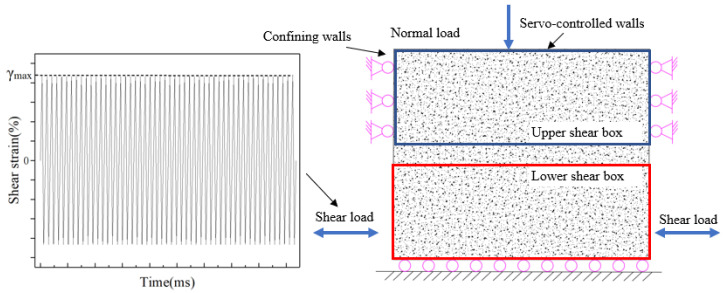
Schematic diagram of the cyclic loading process of the numerical model.

The applied maximum shear strain (γ_max_) follows the periodic (sinusoidal) pattern shown in the left part of Figure 3. By controlling strain, the displacement can be controlled, and then the velocity amplitude (cyclic loading stress amplitude) can be obtained by calculation. From the sine wave in Figure 3, it can be seen that when the maximum strain amplitude reaches the maximum shear strain (γ_max_), then the value remains constant until the cyclic loading stops. Cyclic loading is applied gradually and the strain amplitude can be varied to cover a wide range of strain amplitudes. The use of numerical simulations makes it easy to repeat the test on the same specimen while changing one parameter of the sine wave compared to laboratory tests. Once the specimen (at a specific porosity) is generated and isotopically consolidated, the exact same specimen can be tested numerous times and subjected to various signals without changing the initial conditions.

### 2.4. Comparison of Numerical Simulation and Laboratory Tests

**Figure 4:**
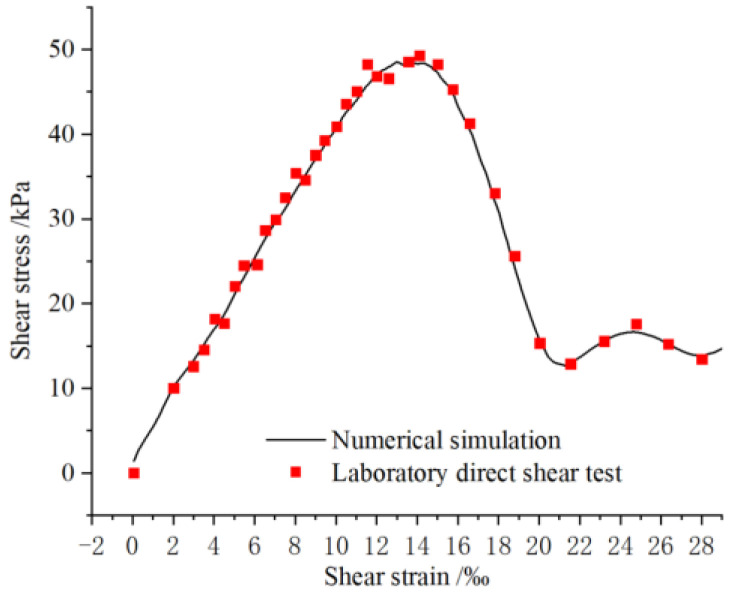
Comparison of straight shear stress-strain curves from laboratory direct shear test and numerical simulation.

The contact and interaction between numerous particles in a PFC model often exhibits macroscopic mechanical behavior. In order to obtain a suitable set of microscopic parameters matching the particles, the calibration of the numerical model is usually tested by comparing the parameters of the macroscopic mechanical behavior of the numerical simulation with the results obtained in the laboratory [46]. The mechanical parameters involved in PFC3D are fine parameters characterizing the properties of the particles, which have a random nature and a complex relationship with the macroscopic mechanical properties, and the calibrated parameters are generally considered reasonable when the obtained macroscopic mechanical properties are consistent with the actual test results through basic mechanical tests [51]. Therefore, based on the macro-mechanical parameters of slip zone soil, the shear stress-strain curve of the slip zone soil is simulated to find and determine the fine-scale parameters of the shear strength of the numerical model corresponding to the appropriate curve. Combined with the recommendations of the work of Hofmann et al. (2015) on the calibration process of the numerical model [52], the numerically simulated mechanical curves that match well with the laboratory tests are obtained, as shown in Figure 4. Table 1 lists the microparameters related to the slip zone soils.

## 3. Analysis for Strain Softening Characteristics of Slip Zone Soil

### 3.1. Coordination Number Analysis for Strain Softening Characteristics 

**Figure 5:**
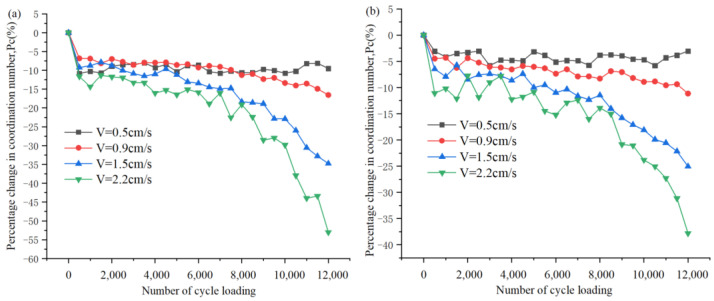

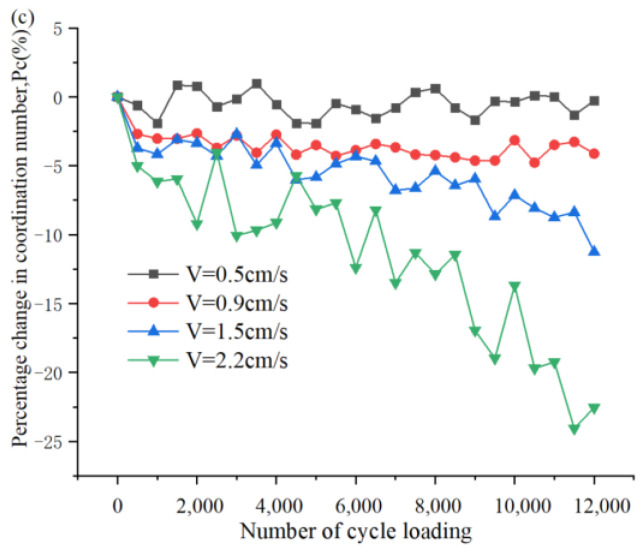
Change of coordination number characterizing the softening process of the slip zone soil: (**a**) 50 kPa; (**b**) 100 kPa; (**c**) 200 kPa.

The coordination number varies widely under different working conditions, and in order to distinguish the degree of variation of the coordination number, the percentage of coordination number is defined as *P_c_*.

(2) $$P_{c}=(C_{n}-C_{ni})/C_{ni}*100$$

where *C_n_* and *C_ni_* are the current and initial coordination numbers in the simulation model, respectively. In the different conditions, the values of *P_c_* of the numerical model are plotted versus the cyclic loading times in Figure 5. In this study, we consider a particle contact breakage rate of 5% (i.e., *P_c_* = −5%) as the maximum limit that can be achieved by particle motion. When the *P_c_* reduction is greater than 5%, significant breakage is assumed. After close observation of Figure 5, when the cyclic loading stress amplitude is 0.5 cm/s, the initial change of *P_c_* does not exceed 10%, which is because this is a small loading rate, which only makes a part of the particles in the numerical soil model move, and even with the increase of cyclic loading, the change of *P_c_* is not significant. Meanwhile, the rate of *P_c_* reduction gradually increased when the cyclic loading stress amplitude increased from 0.9 cm/s to 2.2 cm/s. 

**Figure 6:**
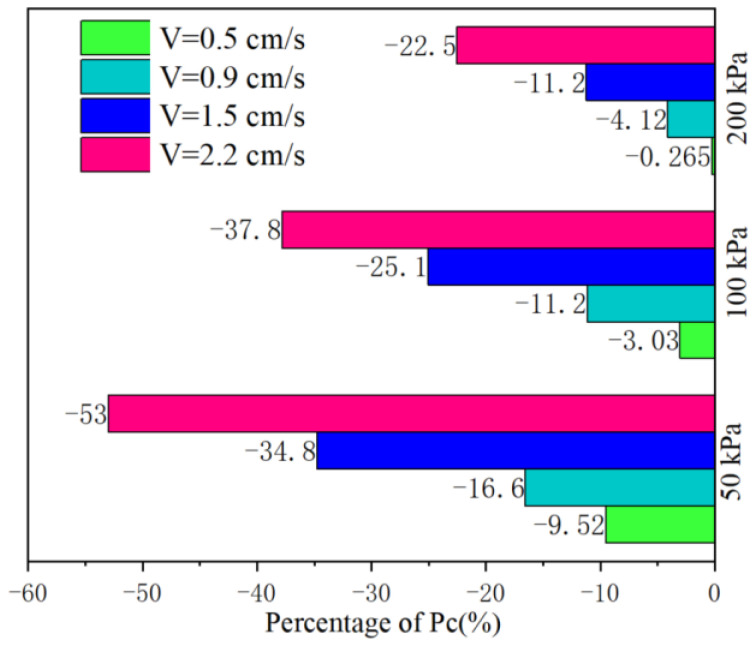
Comparison of the percentage reduction of the coordination number (the number of cyclic loading is 12,000).

In order to highlight the level of strain softening of the slip zone soil material under cyclic loading, when the number of cyclic loading is 12,000, the 100% stacking bar graph in Figure 6 shows the final *P_c_* estimates for the slip zone soil material under different operating conditions, and their values are indicated separately in the corresponding segments. For example, when the slip zone soil material is subjected to a predetermined confining pressure of 50 kPa, at cyclic loading stress amplitudes of 1.5 cm/s and 2.2 cm/s, it can be seen that the strain softening of the slip zone soil corresponds to *P_c_* values of −34.8% and −53.0% in Figure 6. On the other hand, it is obvious from Figure 6 that the cyclic loading stress amplitude is the main factor controlling the number of fractures generated. When observing the effect of different loading stress amplitudes on the *P_c_* of fractures, i.e., the *P_c_* values at different loading stress amplitudes in Figure 5a–c, it is not difficult to find that the change in the fracture is greatest for loading stress amplitude equal to 2.2 cm/s compared with other cyclic loading stresses, which shows that the soil strain softening effect becomes more pronounced with the increase in the number of cyclic loading at larger cyclic loading stresses. This indicates that the cyclic loading stress amplitude plays a deterministic role in the occurrence of fractures (level of strain softening). On the other hand, as the predetermined confining pressure of the slip zone soil increases, i.e., when the confining pressure of soil increases from 50 kPa to 200 kPa (as observed in Figure 5a,c, the number of cyclic loading is 12,000), it is easy to find that when the loading stress amplitude is 0.9 cm/s, the *P_c_* values are −16.6% and −4.12% respectively, and the above discussion reveals that when the confining pressure is reduced from 200 kPa to 50 kPa (the relationship between the confining pressures values is four times), fractures are four times more easily generated in the 50 kPa model than in the 200 kPa model. However, when observing Figure 6, when the cycle loading stress amplitude increase from 0.5 cm/s to 2.2 cm/s (the relationship between the cycle loading stress amplitude values is almost four times), the multiplication of the increase in *P_c_* values is 5.6, 12.5, and 84.9 for the confinement of 50 kPa, 100 kPa, and 200 kPa, respectively. Therefore, we are able to reach the interesting conclusion that although the confining pressure of the slip zone soil can inhibit the increase of fractures, it has a limited inhibitory effect on strain softening, and it is the cyclic loading stress amplitude that is the most key factor in strain softening process of soil.

### 3.2. Stress Analysis for Strain Softening Characteristics of Slip Zone Soil

**Figure 7:**
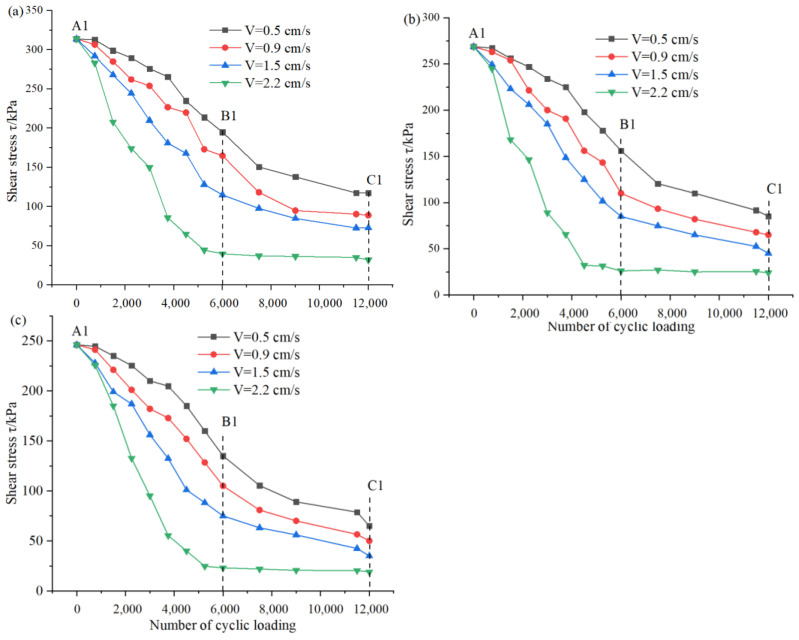
Evolutions of shear stress of slip zone soil under different cycle loading in (**a**) confining pressure = 200 kPa, (**b**) confining pressure = 100 kPa, (**c**) confining pressure = 50 kPa.

**Figure 8:**
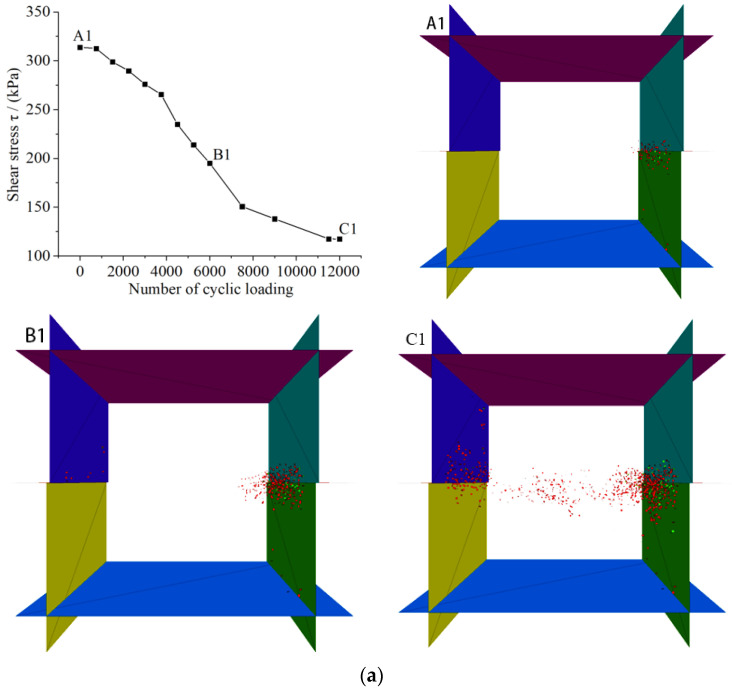

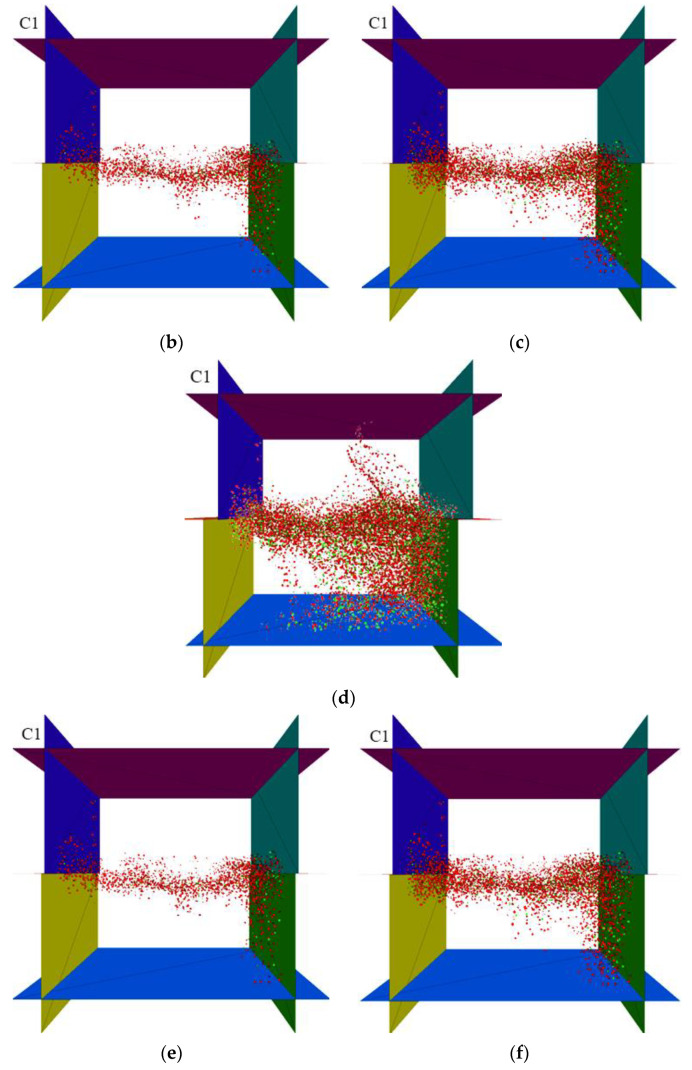

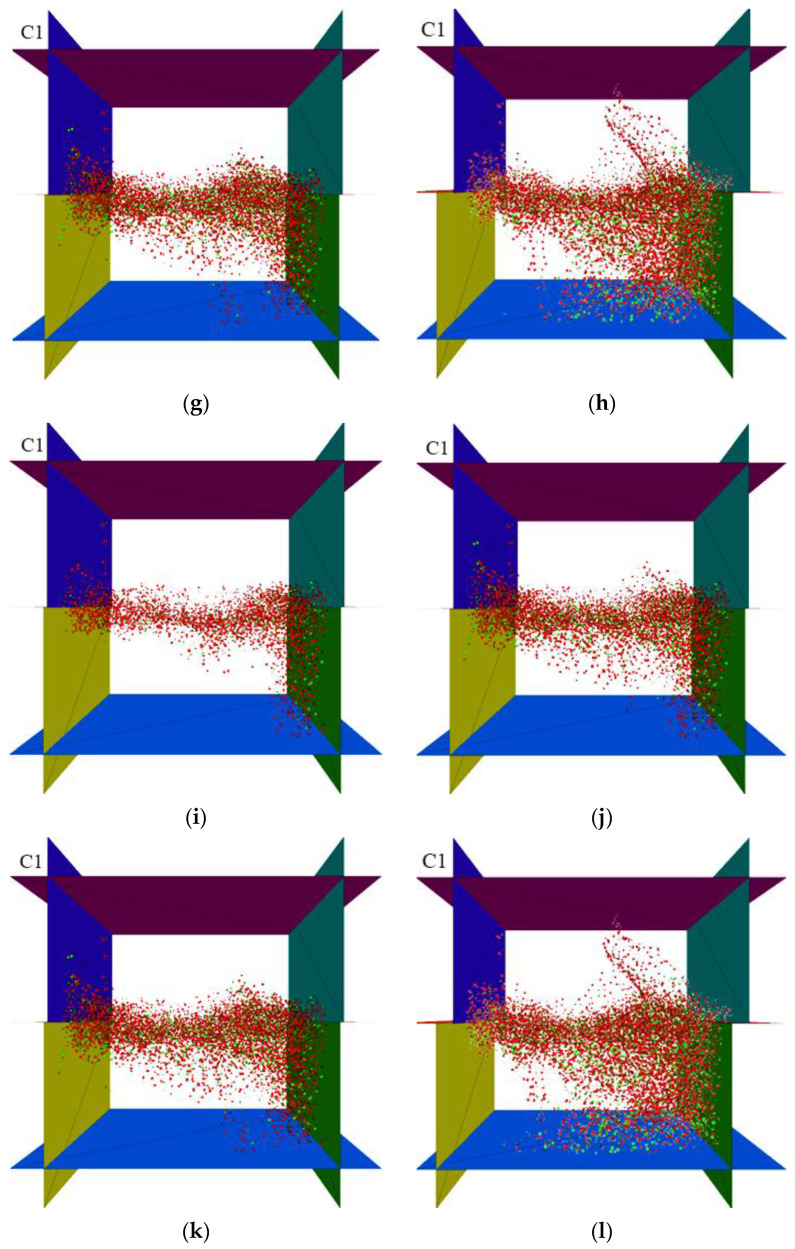
Change of fractures in the numerical model of slip zone soil under different cycle loading in (**a**–**d**) confining pressure = 200 kPa, (**e**–**h**) confining pressure = 100 kPa, and (**i**–**l**) confining pressure = 50 kPa. (**a**) V = 0.5 cm/s, confining pressure = 200 kPa. (**b**) V = 0.9 cm/s, confining pressure = 200 kpa. (**c**) V = 1.5 cm/s, confining pressure = 200 kPa. (**d**) V = 2.2 cm/s, confining pressure = 200 kPa. (**e**) V = 0.5 cm/s, confining pressure = 100 kPa. (**f**) V = 0.9 cm/s, confining pressure = 100 Kpa. (**g**) V = 1.5 cm/s, confining pressure = 100 kPa. (**h**) V = 2.2 cm/s, confining pressure = 100 kPa. (**i**) V = 0.5 cm/s, confining pressure = 50 kPa. (**j**) V = 0.9 cm/s, confining pressure = 50 kPa. (**k**) V = 1.5 cm/s, confining pressure = 50 kPa. (**l**) V = 2.2 cm/s, confining pressure = 50 kPa.

The above discussion reveals that the variation of *C_n_* is an indicative parameter of slip zone soil under the action of cyclic loading and predetermined confining pressure, while the essential cause of the strain softening characteristics of slip zone soil is the variation of shear stress. The variation trend of shear stress of slip zone soil obtained by using the program servo control in numerical simulation, the change law of shear stress of slip zone soil under different predetermined confining pressure and the different number of cyclic loading are discussed, as shown in Figure 7, the change of fractures in the numerical model of slip zone soil is shown in Figure 8, in which red fractures represent tensile fractures and green fractures represent shear fractures, also the information of the numerical model is shown in Figure 2.

From a close view of Figure 7 and Figure 8, it can be seen that the peak shear strength of the slip zone soil material increases with an increase in the predetermined confining pressure, while the cyclic loading stress and the number of cyclic loading also have a positive effect on the strain softening of the slip zone soil. When the cyclic loading stress amplitude is low (less than or equal to 1.5 cm/s), the shear strength decay of the slip zone soil is dominated by the cumulative weakening effect. That is, with the operation of the cyclic loading, the strain softening of the slip zone soil gradually develops and the shear stress gradually decreases, while at a higher cyclic loading amplitude (equal to 2.2 cm/s), the mechanical parameter weakening of the slip zone soil shows the inertial damage characterized by the action of cyclic loading. In this form, there are only two stages of weakening of the slip zone soil mechanical parameters. In the first stage, there is a tendency for rapid decay with the operation of cyclic loading, which means that the slip zone soil is immediately damaged by the loading in this stage. In the second stage, the strength parameters of the slip zone soil have reached the damage limit range, and basically do not change significantly with the increase of the number of cyclic loading. Through the A1 in Figure 8a, it is not difficult to find that fractures appear first at the intersection of the upper and lower shear boxes, which is due to the joint motion of the left upper retaining wall and the right upper retaining wall, which causes the wall to drive the particles attached to the retaining walls on both sides to carry out horizontal motion, thus indirectly transferring them to the center. So based on the diagram of fractures of A1 in Figure 8a, it is easy to know that the first location of the fractures is almost at the junction of the upper and lower shear boxes. When the number of cyclic loading is increased to 6000, the number of fractures appearing at the junction of the shear box further increases, while the location where the fractures exist expands from the junction to the middle of the numerical model, which is caused by the rearrangement and movement of the particles in the middle of the numerical model. Moreover, with the action of cyclic loading, the interaction between the numerical model particles makes more and more particles subject to cyclic loading, and the chain reaction caused by their mutual motion increases geometrically. Therefore, when the number of cyclic loading is 12,000, there is an abrupt increase in the number of fractures in the numerical model of the soil in the slip zone, and a penetrating fracture zone appears inside the model as can be seen by C1 in Figure 8a. The analysis and extension for 200 kPa is applied to 100 kPa and 50 kPa. There is no doubt that the smaller the predetermined confining pressure, the smaller the restraint on the rate of fractures growth, and thus the more pronounced the increase of fractures in the slip zone soil, i.e., the greater the degree of slip zone soil strain softening.

On the other hand, the increase of the cyclic loading stress amplitude also contributes to the penetration of the slip zone soil fractures. From Figure 8a–l, it is not surprising to observe that the greater the cyclic loading stress amplitude, the more obvious the failure damage behavior of the slip zone soil, the more fractures increase, so the larger the macroscopic fracture zone range of the slip zone soil is under the cyclic loading. Interestingly, when the loading stress amplitude is equal to 2.2 cm/s, although the shear stress in the slip zone soil has stabilized after reaching a certain value under different limiting pressures, the number of fractures still increases with the number of cyclic loading. This is because as the number of cyclic loading increases, although the development of fracture is gradually restrained, the cyclic loading causes more particles close to the wall to form a directional arrangement, and the next cyclic loading to be transmitted to the middle of the numerical model needs to pass through particles that have already undergone shear damage behavior. 

Moreover, the residual shear strength of this part of the particles is also the constant friction coefficient between the particles that will continuously consume the energy of the cyclic loading, thus leading to the increase of fractures in the slip zone soil model becomes more and more difficult as the number of cyclic loading increases. Therefore, according to the measurement mechanism of shear stress mentioned above, when the macroscopic fracture zone in the middle part of the slip zone soil model has been formed, the connection between particles far from the interface part becomes more vulnerable to disruption than at the beginning of cyclic loading, so once the larger and dense fracture zone is penetrated, the slip zone soil shear stress values are not significantly correlated with the growth of fractures anymore. That is, landslide occurrence is particularly potent in practical engineering when significant deformation of the slip zone soil occurs due to particle rotation and sliding, followed by a penetrating fracture zone. In general, the development of soil stresses and fractures in the slip zone under different confining pressures and different cyclic loading exhibited a comparable trend of evolution. It is shown that the cyclic loading stress field varies similarly for slip zone soils, and the shear stress decreases monotonically under the cyclic loading. At the same time, penetration of the fracture zone is more likely to occur under higher cycle loading stress and lower confining pressures, as in (Figure 8g,h,k,l). Moreover, after the formation of the macroscopic fracture zone of the slip zone soil, the damage to the strain softening of the slip zone soil by cyclic loading will gradually develop towards the internal changes of the soil. As for A3 in Figure 8, the increase in the number of cyclic loading and the increase in the cyclic stress amplitude will accelerate the strain softening rate of the soil.

## 4. Impacts of Landslide Formation

### 4.1. Mechanical Model of Landslide Mechanism

**Figure 9:**
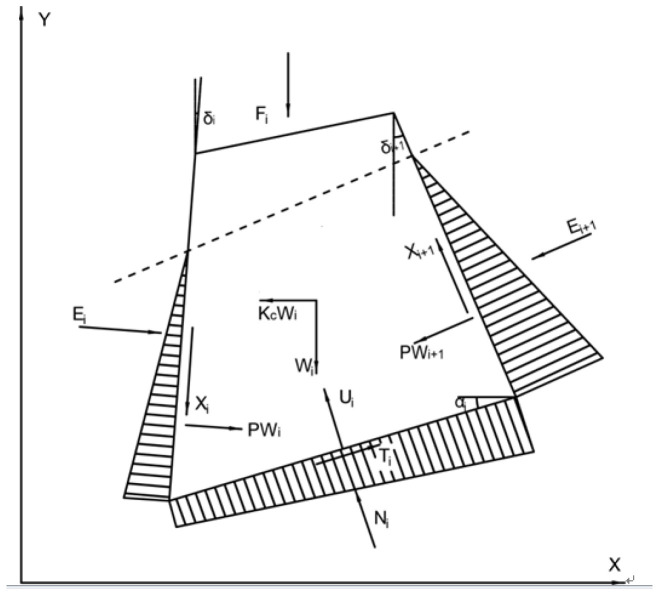
Schematic diagram of the force applied to any block i in the landslide body.

It is well accepted that the trigger mechanism of landslides is reflected by the change in the stability coefficient of the slope. The stability coefficient of the slope is calculated according to the limit equilibrium analysis of the Sarma method, where the change of shear strength is obtained in the previous Section 3.2. Meanwhile, Figure 9 shows the force applied to any block i in the landslide body, which was used for studying the theoretical principle of the Sarma method, the horizontal seismic inertial force is applied to the block, the transient stability factor on the slip zone soil is equal to one.

According to the principle of static balance, the following Equations (2)–(4) are obtained.

(3) ∑X=0, ∑Y=0

(4) $$T_{i}\cos\alpha_{i}-N_{i}\sin\alpha_{i}-K_{c}W_{i}-FX_{i}-X_{i+1}\sin\delta_{i+1}+X_{i}\sin\delta_{i}-E_{i+1}\cos\delta_{i+1}+E_{i}\cos\delta_{i}=0$$

(5) $$T_{i}\sin\alpha_{i}-N_{i}\cos\alpha_{i}-W_{i}+FY_{i}-F_{i}+X_{i+1}\cos\delta_{i+1}-X_{i}\cos\delta_{i}-E_{i+1}\sin\delta_{i+1}+E_{i}\sin\delta_{i}=0\;$$

where *K_c_W_i_* is the horizontal seismic inertial force acting on the block. *T_i_* and *N_i_* are, respectively, the shear force and normal force acting on the bottom surface of the block *i*; *W_i_* is the weight of block *i*; *X_i_* and *X_i_*_+1_ are, respectively, acting on the shear force of the block *i* side and the *i* + 1 th side; is *E_i_* the normal forces acting on the block *i* side, and *E_i_*_+1_ is the normal forces acting on the block *i + 1* side; *F_i_* is the external load acting on the top of the slope; the angle between the block *i* side and the vertical direction is *δ_i_*, the angle between the block *i* + 1 side and the vertical direction is *δ_i_* + 1 the angle between the sliding surface of the block *i* and the horizontal direction is *α_i_*;

According to the Mohr-Coulomb criterion, Equations (5)–(7) can be obtained:

(6) $$T_{i}=(N_{i}-U_{i})\tan\phi_{Bi}+c_{Bi}b_{i}\sec\alpha_{i}$$

where *B_i_* is the width acting on the bottom surface of block *i*; *U_i_* is the water pressure acting on the bottom surface of block *i*, and *φ_Bi_*, *C_Bi_* is the shear strength parameter of the bottom surface of block *i*.

(7) $$X_{i}=(E_{i}-PW_{i})\tan\phi_{Si}+c_{Si}d_{i}$$

(8) $$X_{i+1}=(E_{i+1}-PW_{i+1})\tan\phi_{Si+1}+c_{Si+1}d_{i+1}$$

where *d_i_* is the length of the block i side, *d_i_*_+1_ is the length of the block *i* + 1 side; *PW_i_* is the water pressure acting on the side of the block  i, and *PW_i+1_* is the water pressure acting on the side of the block *i* + 1, *φ_Si_*, *C_Si_* are the shear strength parameter of the block *i* side. Different horizontal seismic inertial forces are calculated through different stability coefficients. When the horizontal seismic inertial force acting on the block is equal to 0, the stability coefficient of the slope is the slope stability coefficient in the natural state.

### 4.2. Calculation of Stability Coefficient

**Figure 10:**
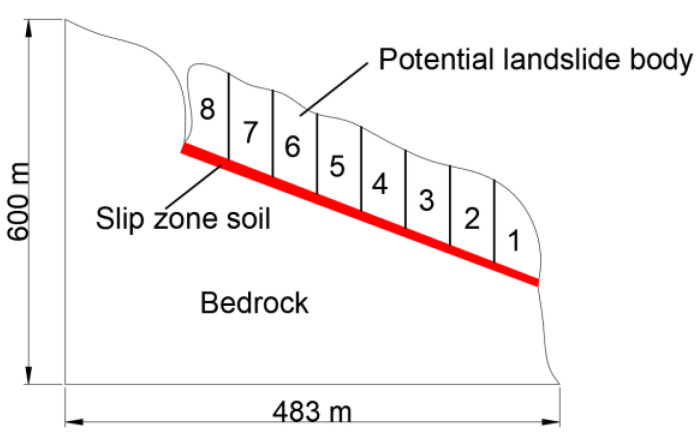
Schematic diagram of section of slope with the slip zone soil.

The section of the slope with slip zone soil is selected as the research section, as shown in Figure 1, in which it is assumed that the slope is a laminated structure, the thickness of the slip zone soil is relatively uniform, and the shear strength parameters of the soil material in the slip zone at different places are the same. In order to reasonably analyze the relationship between the shear strength of the slip zone soil and slope stability, the change of slope stability is discussed and analyzed under different numbers and stress amplitude of cyclic loading. Figure 10 shows a schematic diagram of a section of the slope with the slip zone soil. The sliding section examined includes the slip zone soil, the unloading boundary line and the bedrock. The potential landslide body is divided into strips, and the potential landslide body is divided into eight vertical strips. The cohesion of the potential landslide body and bedrock is 466 KPa and the internal friction angle is 29°, meanwhile, the cohesion between strips from bar 1 to bar 8 is 400 KPa and the internal friction angle is 25°. To simplify the calculation process, the strength parameters of bars 1–8 adopt the uniformity value, the strength parameters of the slip zone soil are the data obtained from the above Section 3.2.

**Figure 11:**
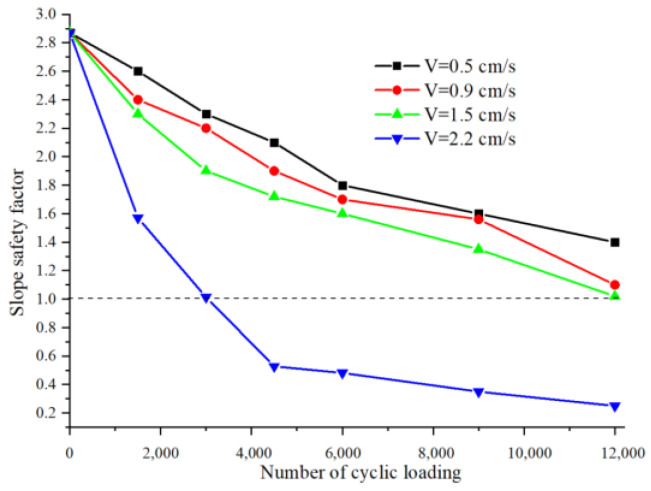
Change of slope safety factor.

According to the results obtained by analyzing the slope stability, the change of slope safety factor is statistically analyzed under different loading numbers and different loading stress amplitude, whose change is shown in Figure 11 and Table 2. 

The stability coefficient of the slope decreases with the increase of stress amplitude and the number of cyclic loading, as can be seen in Figure 11. The calculation in Section 3.2 shows that the strain of the slip zone soil gradually softens under the cyclic loading. The change of shear stress is not only caused by the confining pressure of soil but also the cyclic loading stress plays a key role in the expansion of fractures. However, sometimes the gradual decrease of shear stress is not necessarily all the effect of cyclic loadings, such as the continuous erosion of groundwater and the gradual increase of pore water pressure, which will lead to the gradual decrease of shear stress in the slip zone soil. Meanwhile, by a close analysis of Figure 11, the continuous development and extension of fractures lead to the continuous reduction of the landslide anchorage section, and the stability coefficient of the slope is decreasing, which is one of the key driving factors for landslide occurrence.

Therefore, two possible landslide triggering mechanisms are described. For mode 1, by analyzing A1 and A2 in Figure 8a–c,e–g,i–k, the shear stress gradually decreases with the increase in the number of cyclic loading, and the fracture is also gradually expanded into a penetrating fracture zone, that is, the strain softening behavior occurs slowly with cyclic loading. Additionally, the same analysis applies to the stability coefficient of the slope when cyclic loading stress amplitude is less than or equal to 1.5 cm/s in Figure 11. On the other hand, model 2 can be easily obtained by analyzing A3 in Figure 8d,h,l, it can be seen that when the shear stress no longer increases with the number of cyclic loading, the number of fractures still continues to increase, indicating that the landslide has been triggered. Mode 2 shows that when the cyclic loading stress is larger, the shear stress in the slip zone soil tends to decay rapidly as the number of cyclic load loading increases. Therefore, this phenomenon infers that if the amplitude and number of cycle loading stress are large enough, the strain rate of the slip zone soil will gradually accelerate and a large shear fractures zone is likely to occur, followed by a landslide occurrence, which means that the strain softening behavior of slip zone soils is the result of inertial forces, which expand at a very fast development rate, which is also verified by the data in Figure 11 that is equal to 2.2 cm/s.

### 4.3. Application of Mechanical Learning-Based Time Series Analysis to Slope Stability Prediction

Time series analysis is a highly applied branch of probability statistics, with mathematical tools and theories used in many fields, such as finance and economics, meteorology and hydrology, signal processing and mechanical vibration [53,54]. Although the analysis of long-term trends and cyclical fluctuations control the basic style of time series movements, it is after all not the whole picture of time series movements, and it is more reasonable and superior to use the theory of stochastic processes and statistical theory to examine the time series of long-term trends, seasonal variations and other factors that act together. The analysis of time series is based on the theory of stochastic processes and statistical theory, leading to the stochastic analysis of time series. Stochastic time series analysis enables, on the one hand, the creation of mathematical models that more accurately reflect the dynamic dependencies contained in the series and thereby forecast the future of the system and, on the other hand, statistical methods that more accurately reveal the dynamic structure and laws of the system.

Stochastic analysis of time series usually utilizes the Box–Jenkins modeling approach. The steps for modeling using the Box–Jenkins method are as follows.

(1) Calculate the correlation coefficient and the bias correlation coefficient for a sample of the observed series.

The easiest way to determine whether the data is smoothed or not is to use the image and other analyses using the sample autocorrelation function and sample partial autocorrelation function. When we have a sample series x_t_, we can calculate the covariance of the samples as shown in Equations (8)–(12).

(9) $$\bar{\mu }=\frac{\sum_{t=1}^{n}x_{t}}{n}$$

(10) $$s^{2}=\frac{\sum_{t=1}^{n}\left(x_{t}-\bar{\mu })(x_{\left(t\right)}-\bar{\mu }\right)}{n-1}$$

(11) $$\gamma_{k}=1/n\sum_{t=k+1}^{n}\left(x_{t}-\bar{\mu })(x_{\left(t-k\right)}-\bar{\mu }\right)$$

(12) $$\rho_{k}=\frac{\gamma_{k}}{s^{2}}$$

where *n* is the number of sample series,$\bar{\mu }$ is average, $s^{2}$ is variance, $\gamma_{k}$ is covariance, $\rho_{k}$ is sample autocorrelation function.

(13) $$X_{t}=\Phi_{k1}x_{t-1}+\Phi_{k2}x_{t-2}+\ldots +\Phi_{kk}x_{t-k}+u_{t}$$

Each regression coefficient in the autocorrelation function represents the autocorrelation coefficient between *x_t_* and *x_t_*_−*k*_ after excluding the effect of its intermediate variables *x_t_*_−1_, *x_t_*_−2_,..., *x_t_*_−*k*+1_, i.e., the partial autocorrelation function.

Each of the regression coefficients in the autocorrelation function ($\Phi_{kk}$) represents the autocorrelation coefficient between *x_t_* and *x_t_*_−*k*_ after excluding the effect of its intermediate variables *x_t_*_−1_, *x_t_*_−2_,..., *x_t_*_−*k*+1_, i.e., $\Phi_{kk}$ is the partial autocorrelation function.

(2) Pattern recognition: check whether the sequence is a smooth non-white noise sequence. If the series is a white noise series, the modeling is finished; if the series is a non-stationary series, the modeling method of non-stationary time series is used to build an autoregressive model (ARIMA model) or auto-regressive sliding average model (MA model); if the series is a stationary series, an auto-regressive sliding average model (ARMA model) is built.

(3) Initial order and parameter estimation: after the model is identified, the highest order of the model to which it belongs is framed; then the model is fitted and tested from low to high order within the identified type.

There are a number of criteria that can be used to model {εt}. The criteria are mainly based on the following function (13) shown below.

(14) $$\delta \left(p^{\prime }\right)=n\log\left(\hat{\sigma_{p^{\prime }}^{2}}\right)+p^{\prime }g(n)$$

{εt} is the residual of the data *x_t_*, $\delta \left(p^{\prime }\right)$ is a function of constant order, *p*′ is a constant order, and $\left(\hat{\sigma_{p^{\prime }}}\right)$ is an estimate of the variance of the residual obtained when taking order *p*′, which is the Bayesian information criterion (BIC) criterion when *g*(*n*) = log *n*, if the value of $\delta \left(p^{\prime }\right)$ is smaller, then the prediction model is better.

(4) Goodness-of-fit test: different models are compared using the fixed-order method to determine the most suitable model.

(5) Fit test: the selected model is tested for fit and parameters to further determine the most appropriate model from the selected model.

(6) Prediction: using the prediction model developed, the data is then predicted.

The order in the model identification process was first set to 1, and then the ARIMA (1,1,1)(1,1,1) model was established, and the display model fit measure was selected through Equations (1)–(6), which ultimately allowed the parameter estimate value and standard BIC values to be obtained, as shown in Table 3 and Table 4, and the most appropriate model was selected to determine the most appropriate model by obtaining estimates of the model parameters, and the probabilities of the statistics of the independent variables.

As can be seen from Table 3, the parameter estimate for AR(1) is 0.019 and the probability of the data statistic is 0.762, which means that the original hypothesis, that AR(1) is zero, is accepted. The parameter estimate for MA(1) is 0.714 and the probability of the data statistic is 0, which rejects the original hypothesis that MA(1) is zero. So, the model is not optimal and the analysis of the data is not very appropriate. Therefore, the AR model was selected for another adjustment to obtain the most appropriate model. After the analysis in Table 3 and Table 4 above, it is clear that the data is a non-stationary time series, so the goodness of fit of the ARIMA(1,1,0)(0,1,1) model is established, including all values of the goodness of fit adjusted for R-Square, normalized BIC, etc. The standard BIC value of 8.160 can be seen in Table 5, which is somewhat smaller than the standard BIC value of the ARIMA(1,1,1)(1,1,1) model. At the same time, the probability of the data statistic is 0.848.

**Figure 12:**
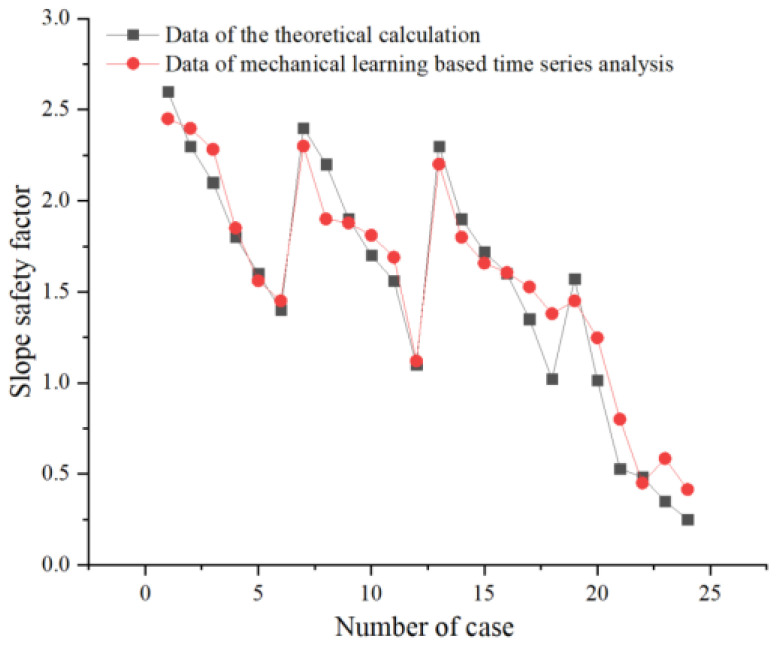
Mechanical learning-based time series analysis to slope stability prediction.

Therefore, this model is a suitable model, so the data in Table 2 are modeled and predicted according to this validated model, as shown in Figure 12. It is easy to see through Figure 12 that the stability coefficients of slopes under different load cases are well predicted, while the difference error between the two is very small. This model can be used for the prediction of stability coefficients of slopes, and also provides an accurate and efficient mechanical learning method for stability analysis of slopes under cyclic loading.

## 5. Conclusions

For rocky slopes containing weak structural surfaces at specified dips, the theory of anchored sections in the slip zone is widely mentioned, where the continuous development and extension of fractures lead to the continuous reduction of the landslide anchorage section (as shown in Figure 7, Figure 8, Figure 9, Figure 10 and Figure 11), which is considered to be a key factor in the occurrence of landslides. The quantitative examination of the correlation between fracture and shear stress will allow us to infer the evolution of landslide occurrence with greater confidence. From this perspective, we developed a three-dimensional slip zone soil numerical model in this study, which aims to incorporate the intermediate mechanism of strain softening. However, it is particularly important to note that we propose a mechanical learning method that can be used to predict stability coefficients for slopes where slopes with predetermined shear planes are subjected to cyclic seismic loads under undrained conditions. 

Another important insight into the microfracture mechanism comes from the comparison of shear stress and *C_n_* in slip zone soils. When the cyclic loading stress amplitude is large, the slip zone soil will be weakened rapidly, immediately after the shear stress no longer changes significantly, but the number of fractures in the slip zone soil still increase. Meanwhile, the gradual change of confining pressure of the slip zone soil rather gently promotes the growth of microfractures. Nevertheless, there are two possible effects associated with sharp changes in the shear stress of slip zone soils: cumulative progressive damage and significant inertial damage. In the second case, the strain softening of the slip zone soils can be explained by the rearrangement of the particles.

Based on the simulation results, possible field observations are illustrated in the context of landslide occurrence, which evolves from changes in the stability coefficient of the slope. The change of stability coefficients of the slope is compared with the development of fractures in the slip zone soil, and two possible landslide triggering mechanisms have been inferred. The strain of the slip zone soil gradually softens under cyclic loading. The shear stress gradually decreases with increasing confining stress, while cyclic loading stress plays a key role in the crack extension. The continuous development and extension of the fracture lead to the continuous reduction of the stability coefficient of the slope, which is another key driving factor for the occurrence of landslides. When the cyclic loading stress is larger, the shear stress of the slip zone soil tends to decay rapidly with the increase in the number of cyclic loadings.

The mechanical learning model proposed in this paper can be used for the prediction of stability coefficients of slopes, and also provides an accurate and efficient mechanical learning method for stability analysis of slopes under cyclic loading. Furthermore, it has to be highlighted once again that the stability of slopes is influenced by a number of factors, including drainage conditions, slope stress states, other forms of dynamic loading, etc. Therefore, the types of slopes mentioned in this paper are actually limited to the layered rock landslides with predetermined shear planes subjected to seismic loading in undrained conditions, which is only a small part of the complex slope stability phenomena.

## Figures and Tables

**Table 1:** Calibrated microscopic parameters of PFC3D particles.

Microparameter of Slip Zone Soil	Unit	Value
Minimum particle radius, *R_min_*	mm	1
Maximum particle radius, *R_max_*	mm	2
Density, ρ	g/cm^3^	2.36
Particle-particle contact modulus, *Ec*	GPa	0.03
Friction coefficient, μ	-	1
Particle normal stiffness to shear stiffness, *kn*/*ks*	-	1.3
Parallel bond normal to shear stiffness ratio, $\bar{kn}/\bar{ks}$	-	1.0
Parallel bond friction angle, $\bar{\varphi }$	deg	25
Parallel bond connection modulus, $\bar{E}$	GPa	0.01
Parallel bond tensile strength $\bar{\sigma_{c}}$	MPa	16.5
Parallel bond cohesion $\bar{C}$	MPa	16.5

**Table 2:** Change of slope safety factor under different loading number and different loading stress amplitude.

Number of Case	Loading Stress Amplitude/(cm/s)	Loading Number	Slope Safety Factor	Number of Case	Loading Stress Amplitude/(cm/s)	Loading Number	Slope Safety Factor
0	0.5	0	2.87	13	1.5	1500	2.3
1	0.5	1500	2.6	14	1.5	3000	1.9
2	0.5	3000	2.3	15	1.5	4500	1.72
3	0.5	4500	2.1	16	1.5	6000	1.6
4	0.5	6000	1.8	17	1.5	9000	1.35
5	0.5	9000	1.6	18	1.5	12,000	1.02
6	0.5	12,000	1.4	19	2.2	1500	1.57
7	0.9	1500	2.4	20	2.2	3000	1.01
8	0.9	3000	2.2	21	2.2	4500	0.52
9	0.9	4500	1.9	22	2.2	6000	0.48
10	0.9	6000	1.7	23	2.2	9000	0.35
11	0.9	9000	1.56	24	2.2	12,000	0.25
12	0.9	12,000	1.1				

**Table 3:** Statistics of the parameter estimate value and probability of the data statistic for ARIMA (1,1,1)(1,1,1) model.

Type of Model		Parameter Estimate Value	Probability of the Data Statistic
Constant		0.227	0.616
AR	Lag1	0.019	0.762
Difference		1	
MA	Lag1	0.714	0.000

**Table 4:** Statistics of the Stationary R-squared and normalized BIC value.

Type of Model	Stationary R-Squared	Normalized BIC	Sratistics	Probability of t Data Statistic
AR	0.425	10.16	2.526	0.748
MA	0.225	12.16	3.526	0.648

**Table 5:** Statistics of the Stationary R-squared and normalized BIC value for ARIMA(1,1,0)(0,1,1) model.

Stationary R-Squared	Normalized BIC	Sratistics	Probability of the Data Statistic
0.747	8.16	9.526	0.848
